# Phylogenomic position of eupelagonemids, abundant, and diverse deep-ocean heterotrophs

**DOI:** 10.1093/ismejo/wrae040

**Published:** 2024-03-08

**Authors:** Gordon Lax, Noriko Okamoto, Patrick J Keeling

**Affiliations:** Department of Botany, University of British Columbia, Vancouver, BC, V6T 1Z4, Canada; Department of Botany, University of British Columbia, Vancouver, BC, V6T 1Z4, Canada; Hakai Institute, Heriot Bay, BC, V0P 1H0, Canada; Department of Botany, University of British Columbia, Vancouver, BC, V6T 1Z4, Canada

**Keywords:** phylogenetics, protist, flagellate, multigene, pelagic

## Abstract

Eupelagonemids, formerly known as Deep Sea Pelagic Diplonemids I (DSPD I), are among the most abundant and diverse heterotrophic protists in the deep ocean, but little else is known about their ecology, evolution, or biology in general. Originally recognized solely as a large clade of environmental ribosomal subunit RNA gene sequences (SSU rRNA), branching with a smaller sister group DSPD II, they were postulated to be diplonemids, a poorly studied branch of Euglenozoa. Although new diplonemids have been cultivated and studied in depth in recent years, the lack of cultured eupelagonemids has limited data to a handful of light micrographs, partial SSU rRNA gene sequences, a small number of genes from single amplified genomes, and only a single formal described species, *Eupelagonema oceanica*. To determine exactly where this clade goes in the tree of eukaryotes and begin to address the overall absence of biological information about this apparently ecologically important group, we conducted single-cell transcriptomics from two eupelagonemid cells. A SSU rRNA gene phylogeny shows that these two cells represent distinct subclades within eupelagonemids, each different from *E. oceanica.* Phylogenomic analysis based on a 125-gene matrix contrasts with the findings based on ecological survey data and shows eupelagonemids branch sister to the diplonemid subgroup Hemistasiidae.

Eupelagonemids are among the most abundant and diverse eukaryotic heterotrophs in the ocean, particularly in the deep ocean, but completely lack the decades or centuries of study that provide other such lineages with biological context illuminating their role in the deep sea ecosystem. They were first recognized from environmental SSU rRNA gene sequences from deep pelagic water [1], and later determined to form two subgroups related to diplonemids (Deep Sea Pelagic Diplonemids, or DSPD I and DSPD II; [2]), arguably the least-studied subgroup of Euglenozoa. Recent research on other diplonemid groups has shown their distribution and ecology is more complex than previously assumed, including a freshwater radiation [3–5]. Likewise the importance of eupelagonemids was not widely recognized until the analysis of TARA-Oceans data showed that DSPD I in particular was extremely abundant and diverse in the deep pelagic ocean, rendering these organisms likely one of the most widespread on the planet [6, 7]. A previous study [8] reported the first light microscopical morphology of DSPD I cells, as well as limited molecular data based on single amplified genomes (SAGs). Subsequently, DSPD I was formally described as Eupelagonemidae, with one described species, *Eupelagonema oceanica* [9].

Despite their prevalence, our knowledge of eupelagonemids remains restricted to these scant data. No strains are in culture, and the vast majority of sequence data is limited to SSU rRNA gene surveys, which do not resolve the placement of eupelagonemids (nor do available SAGs, which had few identifiable genes; [8]). Single-cell transcriptomes have proven a productive strategy to fill such gaps for other uncultivated protists [10–13]. Marine water was collected using a Niskin bottle at 300m depth off the central coast of British Columbia (Station KC10: Lat. 51.6505, Lon. −127.9516) on 3rd July 2022. Several single cells matching the overall description of eupelagonemids were collected and imaged (Supplementary Methods), of which two cells, Eupelagonemid 7 and Eupelagonemid 8, ultimately yielded transcriptomic libraries. Eupelagonemid 7 was oblong in shape, roughly 30μm long and 10μm wide, with an apical papilla protruding ~15 μm from the apical end of the cell (Fig. 1 and Supplementary Files). Eupelagonemid 8 was spindle-shaped, ~25μm long, and 15μm wide, with a round anterior and acute posterior end. When dying, Eupelagonemid 8 released cytoplasm from the round anterior end, suggesting that the cell might have a rigid cell wall with an apical opening. Eupelagonemid 8 has a yellow-brown spherical structure in the posterior region, reminiscent of a digestive vacuole.

**Figure 1:**
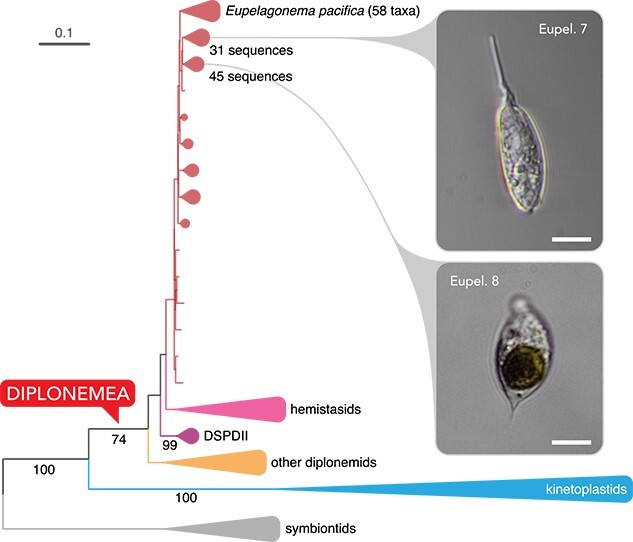
SSU rRNA gene phylogeny of eupelagonemids; ML phylogeny with a comprehensive sampling of diplonemids and two additional euglenozoan outgroups, estimated under GTR + gamma with 1000 non-parametric bootstrap replicates, and major clades are collapsed, for detailed phylogeny, see Supplementary Fig. 1, see online supplementary material for a colour version of this figure, and circles on internodes denote full support (100%); micrographs show cells Eupelagonemid 7 and Eupelagonemid 8, both scale bars are 10 μm.

Single-cell transcriptome libraries were generated using the Smart-Seq3 protocol [14], sequenced on a NextSeq 500 (Illumina) with 2 × 150 bp paired-end reads, and the data assembled and processed (see Supplementary Methods). Out of a public dataset of 240 phylogenetic marker genes, 54 and 36 orthologues were identified from Eupelagonemid 7 and 8, respectively. This is lower than often seen using these methods [10, 12], and another five eupelagonemid cells we isolated yielded even fewer genes (≤10; data not shown). Taken with results from SAGs [8], this may indicate some intrinsic challenges to eupelagonemid genomics, but the current data nevertheless represent roughly a 10-fold increase in phylogenetic marker gene discovery for this group.

To see how Eupelagonemid 7 and 8 represent the diversity of the group, their evolutionary position was first tested within the breadth of full-length SSU rRNA gene sequences from other eupelagonemids. Sequences extracted from transcriptomes (Supplementary Methods) were added to an alignment of 237 eupelagonemids, and other euglenozoans and an unrooted maximum-likelihood (ML) phylogeny representing all major subdivisions of the group was inferred (Fig. 1). Eupelagonemids 7 and 8, and *E. oceanica* all fell within distinct subgroups in different parts of the tree. Eupelagonemids, Hemistasiidae, and DSPD II are all monophyletic (Fig. 1), but none of these groups, nor the branching order between them, is statistically supported. A similar topology was reported previously [15, 16], whereas other SSU rRNA gene analyses show hemistasiids as sister to eupelagonemids and DSPD II [17]. However, in none of these analyses, is the branching order resolved with statistical support, leaving the position of eupelagonemids both contradictory and unsupported.

To resolve this problem, we used the single-cell transcriptome assemblies to conduct multigene phylogenomic analyses. Amino acid sequences for all putative protein-coding genes were inferred from both assemblies, and these were added, along with an additional 12 other new euglenozoans (Supplementary Table 1) to PhyloFisher [18] to infer multigene trees. Ten published eupelagonemid SAGs [8] were initially included as well, but their coverage was too low to be informative. The resulting protein matrix with 9207 and 5856 amino acid sites (54 and 36 genes) for Eupelagonemid 7 and Eupelagonemid 8, respectively, was used to generate a 125-gene, 32 780-site LG + C60 + F + G ML phylogeny of 33 taxa of euglenozoans and outgroups (Fig. 2). The two Eupelagonemids form a clade with full support, and with full support branch sister to the hemistasids. Although this conflicts with the topology of our SSU rRNA gene phylogeny (Fig. 1), it is congruent with some previous reports [18, 19], and none of the published SSU rRNA gene trees are supported. To robustly test our placement of eupelagonemids in the multigene framework, an additional phylogeny under LG + C60 + F + G using only the 19 genes that are available in both Eupelagonemid 7 and 8 (4913 sites total) resulted in the same topology but lower support for the monophyly of hemistasiids (93%; Supplementary Fig. 2, see online supplementary material for a colour version of this figure.). An Approximately Unbiased (AU) test comparing this topology with an alternative found in some bootstrap trees (eupelagonemids branch within hemistasids), rejected the latter in both datasets (see Supplementary Methods).

**Figure 2:**
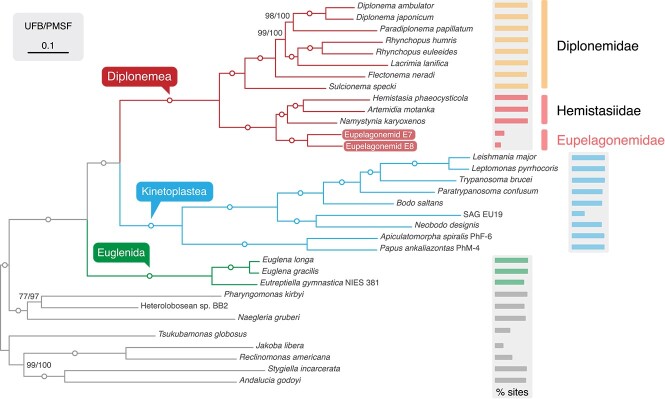
125-gene phylogeny of euglenozoans; ML-phylogeny of 32 780 sites from 33 taxa, including all major subgroups of euglenozoa, except symbiontids (for which there are very limited data available), with heteroloboseans and jakobids as outgroups, and tree estimated under the LG + C60 + F + G model with 1000 ultrafast bootstraps, and 200 non-parametric bootstraps with LG + C60 + F + G + PMSF (PMSF), and circles on internodes denote full support in both analyses; the percentage of sites recovered from each taxon is represented by bars on the right.

Hemistasids are a much less diverse group with only three genera are known to date [17, 19] and environmental sequencing suggesting their diversity is a small fraction of that of eupelagonemids [7]. Hemistasids are also marine, but are only known from surface waters, including coastal habitats, whereas eupelagonemids and DSPD II are predominantly found in deep marine water, and generally open ocean [7, 17]. The diversity of eupelagonemids might therefore represent a radiation from colonizing the deep sea.

Since their discovery, and even their recognition as a major component of the deep sea ecosystem [6, 7], the eupelagonemids have been stubbornly recalcitrant to study. The single-cell transcriptomes described here are the first representative genomic resources for any members of the group and have already redefined their phylogenetic position; placing them sister to another subgroup of diplomenids primarily associated with surface ocean waters. Even though this is an important step, additional data on their biological and ecological functions will require cultures of representative eupelagonemids, along with more depth and breadth of genetic data, and parallel advances for the even more elusive DSPD II group.

## Supplementary Material

supplemental-material_eupelagonemids_v3-1_wrae040

## Data Availability

Raw transcriptome reads are available under NCBI BioProject accession PRJNA1041876, SSU rRNA sequences under accessions OR831206 and OR831207. Assemblies, predicted proteomes, SSU rRNA gene alignment and tree data, and all multigene alignments and trees (single gene and concatenated), and PhyloFisher database folder are deposited under DataDryad accession https://doi.org/10.5061/dryad.hqbzkh1pj.
