# Supplementary material for: Phylogenomic position of eupelagonemids, abundant, and diverse deep-ocean heterotrophs
Source: ISME J. 2024 Mar 8;18(1):wrae040. doi: 10.1093/ismejo/wrae040 (PMC10987973; doi:10.1093/ismejo/wrae040)
Supplement: supplemental-material_eupelagonemids_v3-1_wrae040 [file supplemental-material_eupelagonemids_v3-1_wrae040.pdf]

SUPPLEMENTARY MATERIAL

Methods

Cell isolation and imaging

Seawater was collected with a Niskin bottle from 300 m depth at station KSC10 (Lat. 51.6505, Lon. -127.9516; Calvert Island, British Columbia, Canada) on July 3rd 2022. The water was concentrated approximately ten times on a Pall Supor-800 filter via gravity filtration. The cells were manually isolated from the concentrated seawater with a microcapillary and imaged on a Leica DMIL-LED inverted microscope equipped with a Sony alpha7S III camera at 630X magnification.

Single-cell transcriptomics and assembly

The isolated cells were rinsed three times in drops of clean seawater filtered with an Acrodisc Syringe Filter with a 0.2 µm Pall Supor Membrane, and dispensed into 2 µl of Smart-seq3 lysis buffer. The cDNA was generated using Smart-seq3 with 24 PCR-cycles for cDNA amplification [1]. Libraries were prepared with Illumina DNA library prep and sequenced on a NextSeq 500 (Illumina) platform with 2x150 bp paired end reads by the UBC Sequencing and Bioinformatics Consortium.

Raw reads were read-corrected with rcorrector version 1.0.5 [2], adapter- and quality-trimmed with trimmomatic version 0.39 [3] using parameters ILLUMINACLIP: 2:30:10 LEADING:5 SLIDINGWINDOW:5:16 MINLEN:60, with the following sequences trimmed: Transposase1 (5 ’

CTGTCTCTTATACACATCTCCGAGCCCACGAGAC 3’), Transposase2RC (5 ’ CTGTCTCTTATACACATCTGACGCTGCCGACGA 3’), SmartSeq3_TSO_N8 (5 ’ AGAGACAGATTGCGCAATGNNNNNNNNGGG 3’), SmartSeq3_oligo-dT (5 ’ ACGAGCATCAGCAGCATACGATTTTTTTTTTTTTTTTTTTTTTTTTTTTTTT 3’). The trimmed reads were then assembled with rnaSPAdes version 3.15.5 with default parameters [4]. Protein-coding sequences were predicted with transdecoder version 5.5.0 [5].

Phylogenetics – SSU rDNA

SSU rDNA sequences were extraced from the assemblies using barrnap version 0.9 (https://github.com/tseemann/barrnap) and results blasted against NCBI GenBank’s nr/nt database to distinguish diplonemid SSU rDNAs from contaminants. Identified diplonemid sequences were then aligned with 237 additional sequences of other diplonemids, kinetoplastids, and symbiontids, with a focus on comprehensive sampling of diplonemid sequence diversity (all sequences listed in Supplementary Table 2). This dataset was aligned with MAFFT E-INS-I version 7.481 [6], and trimmed with BMGE version 1.12 [7], yielding a 1,068 bp alignment. A Maximum likelihood (ML) analysis was carried out with RAxML-NG version 1.1.0 [8] under the GTR+GAMMA model and 1,000 non-parametric bootstraps.

Phylogenetics – multigene

To generate a multigene dataset, predicted proteomes of both cells were used as input into phylofisher version 1.2.6 [9]. We also added nine diplonemid and three kinetoplastid taxa (*Hemistasia phaeocysticola*, *Artemidia motanka*, *Namystinia karyoxenos*, *Lacrimia lanifica*,

Rhynchopus humris, *R. euleeides*, *Diplonema japonicum*, *Paradiplonema papillatum*, *Sulcionema specki*, *Papus ankaliazontas*, *Apiculatamorpha spiralis,* SAG EU19). After checking each of the 240 single gene trees for contaminant, paralogous, or otherwise aberrant sequences, we recovered 11.85% of sites for Eupelagonemid 7 (54 genes), and 7.54% of sites for cell Eupelagonemid 8 (36 genes) out of a total of 77,659 sites (240 genes).

A final concatenated dataset of 125 genes from 33 euglenozoan taxa and outgroups (Heterolobosea, Jakobida, and *Tsukubamonas*, see Supplementary Table 1 for a full list of taxa included and their gene and site coverages) with 32,780 sites (site coverage with this gene selection was 28.1% for Eupelagonemid 7 and 17.9% for Eupelagonemid 8) was used to run a ML-phylogeny using IQ-TREE2 version 2.2.0 [10] under the LG+C60+F+G model with 1,000 ultrafast bootstraps (UFB [11]). We additionally ran the same dataset under a posterior mean site frequency model (PMSF [12]) with 200 non-parametric bootstraps, using the previous LG+C60+F+G tree as a guide tree. We also generated a dataset that had higher coverage for eupelagonemids, using only the 28 genes that are shared between cell Eupelagonemid 7 and 8, and ran this dataset with IQ-TREE2 under the LG+C60+F+G model with 1,000 UFB. We also ran constrained tree searches for both 125 and 28 genes using the topology recovered in Figure 1 (eupelagonemids sister to hemistasiids), and an alternative topology found in around 7% of bootstrap trees of the 28-gene analysis (Supplementary Figure 2; eupelagonemids branching within hemistasiids). An Approximately-Unbiased test [13] in IQ-TREE2 comparing these topologies favoured topology 1 in the 28-gene dataset (0.601 vs 0.139), and rejected the alternative topology 2 in the 125-gene dataset (0.511 vs 0).

References

1. Hagemann-Jensen M, Ziegenhain C, Chen P, Ramsköld D, Hendriks G-J, Larsson AJM, et al. Single-cell RNA counting at allele and isoform resolution using Smart-seq3. *Nat Biotechnol* 2020; **38**: 708–714.

2. Song L, Florea L. Rcorrector: efficient and accurate error correction for Illumina RNA-seq reads. *GigaScience* 2015; **4**: 1–8.

3. Bolger AM, Lohse M, Usadel B. Trimmomatic: a flexible trimmer for Illumina sequence data. *Bioinformatics* 2014; **30**: 2114–2120.

4. Bushmanova E, Antipov D, Lapidus A, Prjibelski AD. rnaSPAdes: a de novo transcriptome assembler and its application to RNA-Seq data. *bioRxiv* 2018; **32**: 1009–1015.

5. Grabherr MG, Haas BJ, Yassour M, Levin JZ, Thompson DA, Amit I, et al. Full-length transcriptome assembly from RNA-Seq data without a reference genome. *Nat Biotechnol* 2011; **29**: 644–652.

6. Katoh K, Standley DM. MAFFT multiple sequence alignment software version 7: improvements in performance and usability. *Mol Biol Evol* 2013; **30**: 772–780.

7. Criscuolo A, Gribaldo S. BMGE (Block Mapping and Gathering with Entropy): a new software for selection of phylogenetic informative regions from multiple sequence alignments. *BMC Evol Biol* 2010; **10**.

8. Kozlov AM, Darriba D, Flouri T, Morel B, Stamatakis A. RAxML-NG: A fast, scalable, and user-friendly tool for maximum likelihood phylogenetic inference. *Bioinformatics* 2019; **35**: 4453–4455.

9. Tice AK, Žihala D, Pánek T, Jones RE, Salomaki ED, Nenarokov S, et al. PhyloFisher: A phylogenomic package for resolving eukaryotic relationships. *PLoS Biol* 2021; **19**: e3001365.

10. Minh BQ, Schmidt HA, Chernomor O, Schrempf D, Woodhams MD, Haeseler A von, et al. IQ-TREE 2: New models and efficient methods for phylogenetic inference in the genomic era. *Mol Biol Evol* 2020; **37**: 1530–1534.

11. Minh BQ, Nguyen MAT, Haeseler A von. Ultrafast approximation for phylogenetic bootstrap. *Mol Biol Evol* 2013; **30**: 1188–1195.

12. Wang H-C, Minh BQ, Susko E, Roger AJ. Modeling site heterogeneity with Posterior Mean Site Frequency profiles accelerates accurate phylogenomic estimation. *Syst Biol* 2018; **67**: 216–235.

13. Shimodaira H. An Approximately Unbiased test of phylogenetic tree selection. *Syst Biol* 2002; **51**: 492–508.

SUPPLEMENTARY LEGENDS

Supplementary Figure S1. SSU rDNA phylogeny of eupelagonemids, including other euglenozoans. Maximum-likelihood phylogeny with a comprehensive sampling of diplonemids, kinetoplastids, and symbiontids, estimated under the GTR+Gamma model with 1,000 non-parametric bootstrap replicates. Circles on internodes denote full support (100%). This phylogeny is identical to Figure 1 but without collapsed clades.

Supplementary Figure S2. Multigene ML-phylogeny of eupelagonemids, using a 28-gene, 4,913-site dataset from 33 taxa, including major euglenozoan subgroups, except symbiontids. Estimated in IQ-TREE2 under the LG+C60+F+G model with 1,000 ultrafast bootstraps. Circles on internodes depict full (100%) UFB support.

Supplementary Table S1. Taxa used in multigene analyses, with numbers of genes, percentage of sites covered, and source.

Supplementary Table S2. Taxa used in SSU rDNA tree, with accession numbers and lengths.

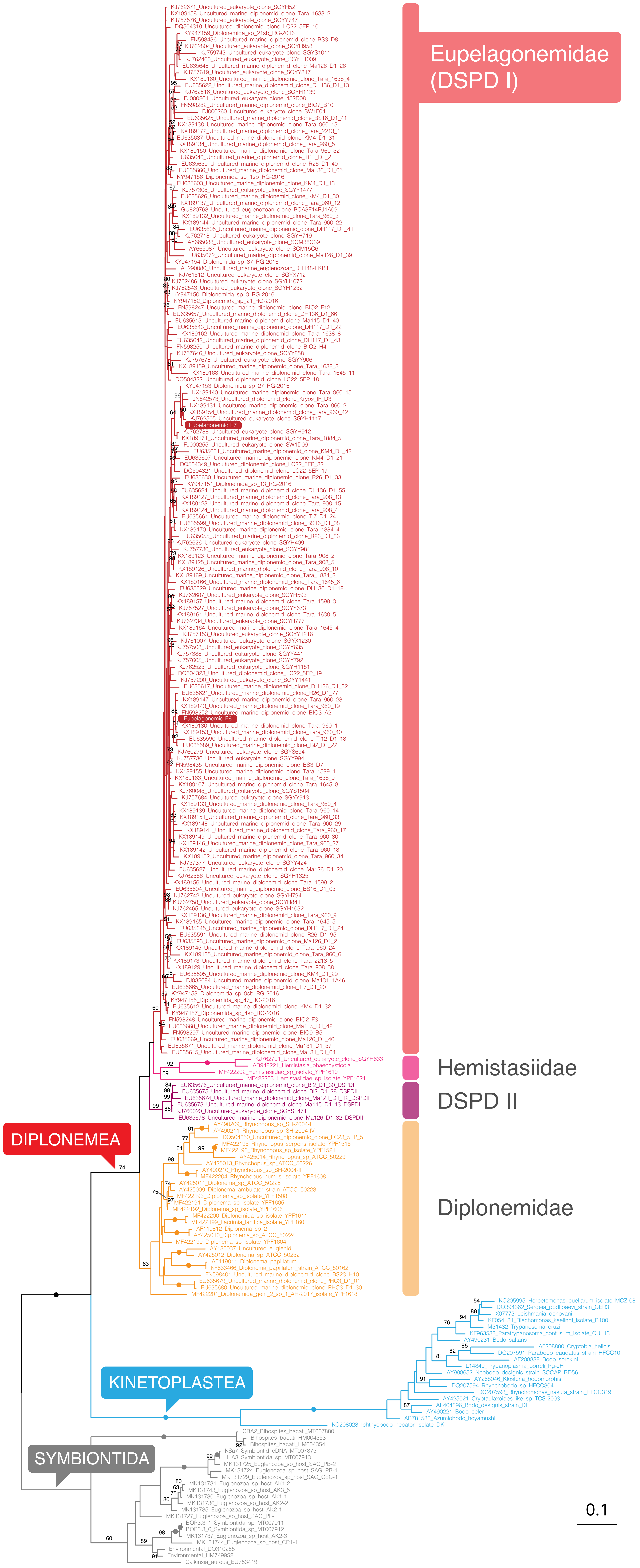

Eupelagonemidae (DSPD I)
KJ762671_Uncultured_eukaryote_clone_SGYH521
KX189158_Uncultured_marine_diplonemid_clone_Tara_1638_2
KJ757576_Uncultured_eukaryote_clone_SGYY747
DQ504319_Uncultured_diplonemid_clone_LC22_5EP_10
KY947159_Diplonemida_sp_21sb_RG-2016
FN598436_Uncultured_marine_diplonemid_clone_BS3_D8
KJ762804_Uncultured_eukaryote_clone_SGYH958
KJ759743_Uncultured_eukaryote_clone_SGYS1011
KJ762460_Uncultured_eukaryote_clone_SGYH1009
EU635648_Uncultured_marine_diplonemid_clone_Ma126_D1_26
KJ757619_Uncultured_eukaryote_clone_SGYY817
KX189160_Uncultured_marine_diplonemid_clone_Tara_1638_4
EU635622_Uncultured_marine_diplonemid_clone_DH136_D1_13
KJ762516_Uncultured_eukaryote_clone_SGYH1139
FJ000261_Uncultured_eukaryote_clone_452D08
FN598282_Uncultured_marine_diplonemid_clone_BIO7_B10
FJ000260_Uncultured_eukaryote_clone_SW1F04
EU635625_Uncultured_marine_diplonemid_clone_BS16_D1_41
KX189138_Uncultured_marine_diplonemid_clone_Tara_960_13
KX189172_Uncultured_marine_diplonemid_clone_Tara_2213_1
EU635637_Uncultured_marine_diplonemid_clone_KM4_D1_31
KX189134_Uncultured_marine_diplonemid_clone_Tara_960_5
KX189150_Uncultured_marine_diplonemid_clone_Tara_960_32
EU635640_Uncultured_marine_diplonemid_clone_Ti11_D1_21
EU635639_Uncultured_marine_diplonemid_clone_R26_D1_40
EU635666_Uncultured_marine_diplonemid_clone_Ma136_D1_05
KY947156_Diplonemida_sp_1sb_RG-2016
EU635603_Uncultured_marine_diplonemid_clone_KM4_D1_13
KJ757308_Uncultured_eukaryote_clone_SGYY1477
EU635626_Uncultured_marine_diplonemid_clone_KM4_D1_30
KX189137_Uncultured_marine_diplonemid_clone_Tara_960_12
GU820768_Uncultured_euglenozoan_clone_BCA3F14RJ1A09
KX189132_Uncultured_marine_diplonemid_clone_Tara_960_3
KX189144_Uncultured_marine_diplonemid_clone_Tara_960_22
EU635605_Uncultured_marine_diplonemid_clone_DH117_D1_41
KJ762718_Uncultured_eukaryote_clone_SGYH719
AY665088_Uncultured_eukaryote_clone_SCM38C39
AY665087_Uncultured_eukaryote_clone_SCM15C6
EU635672_Uncultured_marine_diplonemid_clone_Ma126_D1_39
KY947154_Diplonemida_sp_37_RG-2016
AF290080_Uncultured_marine_euglenozoan_DH148-EKB1
KJ761512_Uncultured_eukaryote_clone_SGYX712
KJ762486_Uncultured_eukaryote_clone_SGYH1072
KJ762543_Uncultured_eukaryote_clone_SGYH1232
KY947150_Diplonemida_sp_3_RG-2016
KY947152_Diplonemida_sp_21_RG-2016
FN598247_Uncultured_marine_diplonemid_clone_BIO2_F12
EU635657_Uncultured_marine_diplonemid_clone_DH136_D1_66
EU635613_Uncultured_marine_diplonemid_clone_Ma115_D1_40
EU635643_Uncultured_marine_diplonemid_clone_DH117_D1_22
KX189162_Uncultured_marine_diplonemid_clone_Tara_1638_8
EU635642_Uncultured_marine_diplonemid_clone_DH117_D1_43
FN598250_Uncultured_marine_diplonemid_clone_BIO2_H4
KJ757646_Uncultured_eukaryote_clone_SGYY858
KJ757678_Uncultured_eukaryote_clone_SGYY906
KX189159_Uncultured_marine_diplonemid_clone_Tara_1638_3
KX189168_Uncultured_marine_diplonemid_clone_Tara_1645_11
DQ504322_Uncultured_diplonemid_clone_LC22_5EP_18
KY947153_Diplonemida_sp_27_RG-2016
KX189140_Uncultured_marine_diplonemid_clone_Tara_960_15
JN542573_Uncultured_diplonemid_clone_Kryos_IF_D3
KX189131_Uncultured_marine_diplonemid_clone_Tara_960_2
KX189154_Uncultured_marine_diplonemid_clone_Tara_960_42
KJ762505_Uncultured_eukaryote_clone_SGYH1117
Eupelagonemid E7
KJ762788_Uncultured_eukaryote_clone_SGYH912
KX189171_Uncultured_marine_diplonemid_clone_Tara_1884_5
FJ000255_Uncultured_eukaryote_clone_SW1D09
EU635631_Uncultured_marine_diplonemid_clone_KM4_D1_42
EU635607_Uncultured_marine_diplonemid_clone_KM4_D1_21
DQ504349_Uncultured_diplonemid_clone_LC22_5EP_32
DQ504321_Uncultured_diplonemid_clone_LC22_5EP_17
EU635630_Uncultured_marine_diplonemid_clone_R26_D1_33
KY947151_Diplonemida_sp_13_RG-2016
EU635624_Uncultured_marine_diplonemid_clone_DH136_D1_55
KX189127_Uncultured_marine_diplonemid_clone_Tara_908_13
KX189128_Uncultured_marine_diplonemid_clone_Tara_908_15
KX189124_Uncultured_marine_diplonemid_clone_Tara_908_4
EU635661_Uncultured_marine_diplonemid_clone_Ti7_D1_24
EU635599_Uncultured_marine_diplonemid_clone_BS16_D1_08
KX189170_Uncultured_marine_diplonemid_clone_Tara_1884_4
EU635655_Uncultured_marine_diplonemid_clone_R26_D1_86
KJ762626_Uncultured_eukaryote_clone_SGYH409
KJ757730_Uncultured_eukaryote_clone_SGYY981
KX189123_Uncultured_marine_diplonemid_clone_Tara_908_2
KX189125_Uncultured_marine_diplonemid_clone_Tara_908_5
KX189126_Uncultured_marine_diplonemid_clone_Tara_908_10
KX189169_Uncultured_marine_diplonemid_clone_Tara_1884_2
KX189166_Uncultured_marine_diplonemid_clone_Tara_1645_6
EU635629_Uncultured_marine_diplonemid_clone_DH136_D1_18
KJ762687_Uncultured_eukaryote_clone_SGYH593
KX189157_Uncultured_marine_diplonemid_clone_Tara_1599_3
KJ757527_Uncultured_eukaryote_clone_SGYY673
KX189161_Uncultured_marine_diplonemid_clone_Tara_1638_5
KJ762734_Uncultured_eukaryote_clone_SGYH777
KX189164_Uncultured_marine_diplonemid_clone_Tara_1645_4
KJ757153_Uncultured_eukaryote_clone_SGYY1216
KJ761007_Uncultured_eukaryote_clone_SGYX1230
KJ757508_Uncultured_eukaryote_clone_SGYY635
KJ757388_Uncultured_eukaryote_clone_SGYY441
KJ757605_Uncultured_eukaryote_clone_SGYY792
KJ762523_Uncultured_eukaryote_clone_SGYH1151
DQ504323_Uncultured_diplonemid_clone_LC22_5EP_19
KJ757290_Uncultured_eukaryote_clone_SGYY1441
EU635617_Uncultured_marine_diplonemid_clone_DH136_D1_32
EU635621_Uncultured_marine_diplonemid_clone_R26_D1_77
KX189147_Uncultured_marine_diplonemid_clone_Tara_960_28
KX189143_Uncultured_marine_diplonemid_clone_Tara_960_19
FN598252_Uncultured_marine_diplonemid_clone_BIO3_A2
Eupelagonemid E8
KX189130_Uncultured_marine_diplonemid_clone_Tara_960_1
KX189153_Uncultured_marine_diplonemid_clone_Tara_960_40
EU635590_Uncultured_marine_diplonemid_clone_Ti12_D1_18
EU635589_Uncultured_marine_diplonemid_clone_Bi2_D1_22
KJ760279_Uncultured_eukaryote_clone_SGYS694
KJ757736_Uncultured_eukaryote_clone_SGYY994
FN598435_Uncultured_marine_diplonemid_clone_BS3_D7
KX189155_Uncultured_marine_diplonemid_clone_Tara_1599_1
KX189163_Uncultured_marine_diplonemid_clone_Tara_1638_9
KX189167_Uncultured_marine_diplonemid_clone_Tara_1645_8
KJ760048_Uncultured_eukaryote_clone_SGYS1504
KJ757684_Uncultured_eukaryote_clone_SGYY913
KX189133_Uncultured_marine_diplonemid_clone_Tara_960_4
KX189139_Uncultured_marine_diplonemid_clone_Tara_960_14
KX189151_Uncultured_marine_diplonemid_clone_Tara_960_33
KX189148_Uncultured_marine_diplonemid_clone_Tara_960_29
KX189141_Uncultured_marine_diplonemid_clone_Tara_960_17
KX189149_Uncultured_marine_diplonemid_clone_Tara_960_30
KX189146_Uncultured_marine_diplonemid_clone_Tara_960_27
KX189142_Uncultured_marine_diplonemid_clone_Tara_960_18
KX189152_Uncultured_marine_diplonemid_clone_Tara_960_34
KJ757377_Uncultured_eukaryote_clone_SGYY424
EU635627_Uncultured_marine_diplonemid_clone_Ma126_D1_20
KJ762566_Uncultured_eukaryote_clone_SGYH1325
KX189156_Uncultured_marine_diplonemid_clone_Tara_1599_2
EU635604_Uncultured_marine_diplonemid_clone_BS16_D1_03
KJ762742_Uncultured_eukaryote_clone_SGYH794
KJ762758_Uncultured_eukaryote_clone_SGYH841
KJ762465_Uncultured_eukaryote_clone_SGYH1032
KX189136_Uncultured_marine_diplonemid_clone_Tara_960_9
KX189165_Uncultured_marine_diplonemid_clone_Tara_1645_5
EU635645_Uncultured_marine_diplonemid_clone_DH117_D1_24
EU635591_Uncultured_marine_diplonemid_clone_R26_D1_95
EU635593_Uncultured_marine_diplonemid_clone_Ma126_D1_21
KX189145_Uncultured_marine_diplonemid_clone_Tara_960_24
KX189135_Uncultured_marine_diplonemid_clone_Tara_960_6
KX189173_Uncultured_marine_diplonemid_clone_Tara_2213_5
KX189129_Uncultured_marine_diplonemid_clone_Tara_908_38
EU635595_Uncultured_marine_diplonemid_clone_KM4_D1_29
FJ032684_Uncultured_marine_diplonemid_clone_Ma131_1A46
EU635665_Uncultured_marine_diplonemid_clone_Ti7_D1_20
KY947158_Diplonemida_sp_9sb_RG-2016
KY947155_Diplonemida_sp_47_RG-2016
EU635612_Uncultured_marine_diplonemid_clone_KM4_D1_32
KY947157_Diplonemida_sp_4sb_RG-2016
FN598248_Uncultured_marine_diplonemid_clone_BIO2_F3
EU635668_Uncultured_marine_diplonemid_clone_Ma115_D1_42
FN598297_Uncultured_marine_diplonemid_clone_BIO9_B5
EU635669_Uncultured_marine_diplonemid_clone_Ma126_D1_46
EU635671_Uncultured_marine_diplonemid_clone_Ma131_D1_37
EU635615_Uncultured_marine_diplonemid_clone_Ma131_D1_04
Hemistasiidae
KJ762701_Uncultured_eukaryote_clone_SGYH633
AB948221_Hemistasia_phaeocysticola
MF422202_Hemistasiidae_sp_isolate_YPF1610
MF422203_Hemistasiidae_sp_isolate_YPF1621
DSPD II
EU635676_Uncultured_marine_diplonemid_clone_Bi2_D1_30_DSPDII
EU635675_Uncultured_marine_diplonemid_clone_Bi2_D1_28_DSPDII
EU635674_Uncultured_marine_diplonemid_clone_Ma121_D1_12_DSPDII
EU635673_Uncultured_marine_diplonemid_clone_Ma115_D1_13_DSPDII
KJ760020_Uncultured_eukaryote_clone_SGYS1471
EU635678_Uncultured_marine_diplonemid_clone_Ma126_D1_32_DSPDII
DIPLONEMEA
Diplonemidae
AY490209_Rhynchopus_sp_SH-2004-I
AY490211_Rhynchopus_sp_SH-2004-IV
DQ504350_Uncultured_diplonemid_clone_LC23_5EP_5
MF422195_Rhynchopus_serpens_isolate_YPF1515
MF422196_Rhynchopus_sp_isolate_YPF1521
AY425014_Rhynchopus_sp_ATCC_50229
AY425013_Rhynchopus_sp_ATCC_50226
AY490210_Rhynchopus_sp_SH-2004-II
MF422204_Rhynchopus_humris_isolate_YPF1608
AY425011_Diplonema_sp_ATCC_50225
AY425009_Diplonema_ambulator_strain_ATCC_50223
MF422193_Diplonema_sp_isolate_YPF1508
MF422191_Diplonema_sp_isolate_YPF1605
MF422192_Diplonema_sp_isolate_YPF1606
MF422200_Diplonema_sp_isolate_YPF1611
MF422199_Lacrimia_lanifica_isolate_YPF1601
AF119812_Diplonema_sp_2
AY425010_Diplonema_sp_ATCC_50224
MF422190_Diplonema_sp_isolate_YPF1604
AY180037_Uncultured_euglenid
AY425012_Diplonema_sp_ATCC_50232
AF119811_Diplonema_papillatum
KF633466_Diplonema_papillatum_strain_ATCC_50162
FN598401_Uncultured_marine_diplonemid_clone_BS23_H10
EU635679_Uncultured_marine_diplonemid_clone_PHC3_D1_01
EU635680_Uncultured_marine_diplonemid_clone_PHC3_D1_30
MF422201_Diplonemida_gen_2_sp_1_AH-2017_isolate_YPF1618
KINETOPLASTEA
KC205995_Herpetomonas_puellarum_isolate_MCZ-08
DQ394362_Sergeia_podlipaevi_strain_CER3
X07773_Leishmania_donovani
KF054131_Blechomonas_keelingi_isolate_B100
M31432_Trypanosoma_cruzi
KF963538_Paratrypanosoma_confusum_isolate_CUL13
AY490231_Bodo_saltans
AF208880_Cryptobia_helicis
DQ207591_Parabodo_caudatus_strain_HFCC10
AF208888_Bodo_sorokini
L14840_Trypanoplasma_borreli_Pg-JH
AY998652_Neobodo_designis_strain_SCCAP_BD56
AY268046_Klosteria_bodomorphis
DQ207594_Rhynchobodo_sp_HFCC304
DQ207598_Rhynchomonas_nasuta_strain_HFCC319
AY425021_Cryptaulaxoides-like_sp_TCS-2003
AF464896_Bodo_designis_strain_DH
AY490221_Bodo_celer
AB781588_Azumiobodo_hoyamushi
KC208028_Ichthyobodo_necator_isolate_DK
SYMBIONTIDA
CBA2_Bihospites_bacati_MT007880
Bihospites_bacati_HM004353
Bihospites_bacati_HM004354
KSa7_Symbiontid_cDNA_MT007875
HLA3_Symbiontida_sp_MT007913
MK131725_Euglenozoa_sp_host_SAG_PB-2
MK131724_Euglenozoa_sp_host_SAG_PB-1
MK131729_Euglenozoa_sp_host_SAG_CdC-1
MK131731_Euglenozoa_sp_host_AK1-2
MK131743_Euglenozoa_sp_host_AK3_5
MK131730_Euglenozoa_sp_host_AK1-1
MK131736_Euglenozoa_sp_host_AK2-2
MK131735_Euglenozoa_sp_host_AK2-1
MK131727_Euglenozoa_sp_host_SAG_PL-1
BOP3.3_1_Symbiontida_sp_MT007911
BOP3.3_6_Symbiontida_sp_MT007912
MK131737_Euglenozoa_sp_host_AK2-3
MK131744_Euglenozoa_sp_host_CR1-1
Environmental_DQ310255
Environmental_HM749952
Calkinsia_aureus_EU753419
0.1

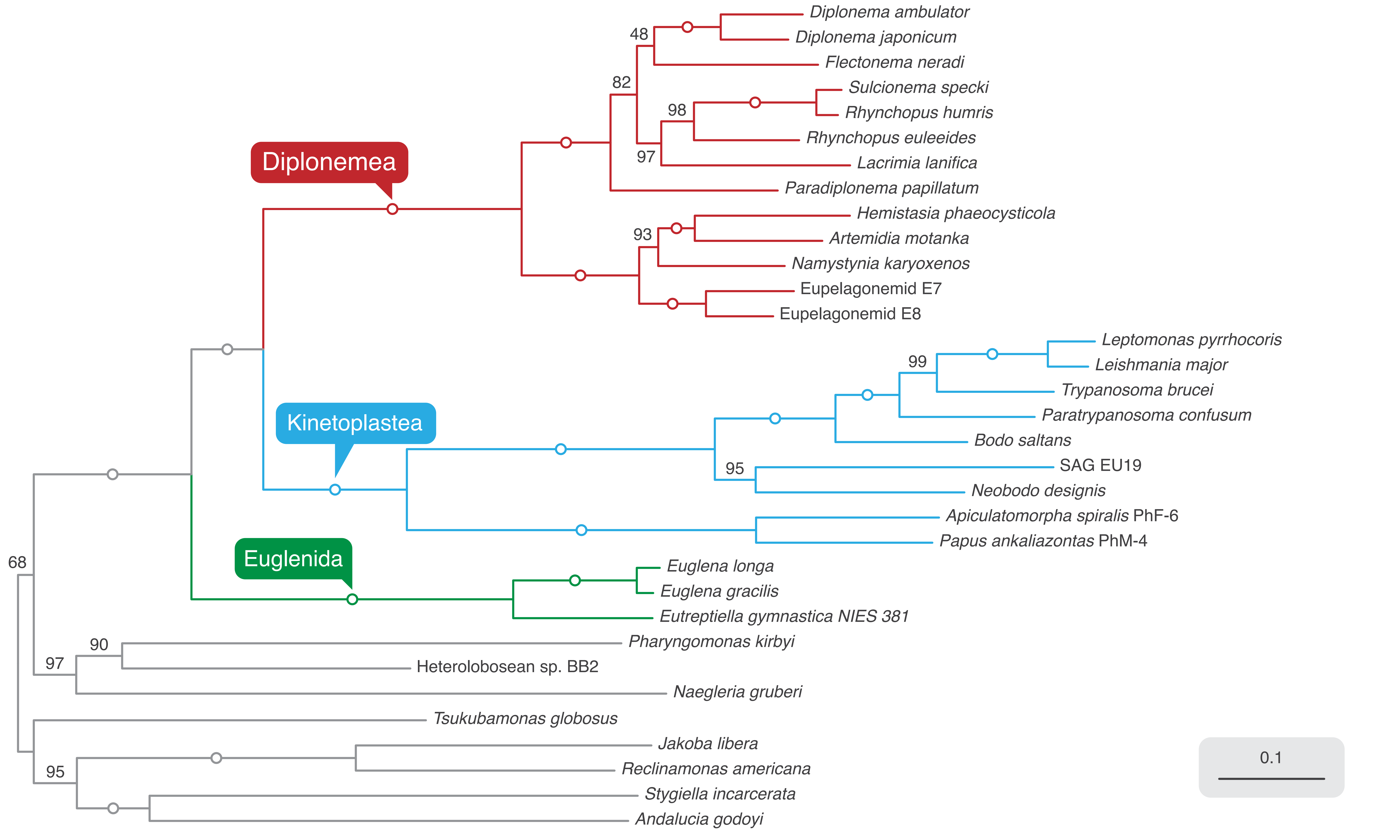
Diplonemea
Kinetoplastea
Euglenida
Diplonema ambulator
Diplonema japonicum
Flectonema neradi
Sulcionema specki
Rhynchopus humris
Rhynchopus euleeides
Lacrimia lanifica
Paradiplonema papillatum
Hemistasia phaeocysticola
Artemidia motanka
Namystynia karyoxenos
Eupelagonemid E7
Eupelagonemid E8
Leptomonas pyrrhocoris
Leishmania major
Trypanosoma brucei
Paratrypanosoma confusum
Bodo saltans
SAG EU19
Neobodo designis
Apiculatomorpha spiralis PhF-6
Papus ankaliazontas PhM-4
Euglena longa
Euglena gracilis
Eutreptiella gymnastica NIES 381
Pharyngomonas kirbyi
Heterolobosean sp. BB2
Naegleria gruberi
Tsukubamonas globosus
Jakoba libera
Reclinamonas americana
Stygiella incarcerata
Andalucia godoyi
48
82
98
97
93
99
95
68
90
97
95
0.1

TABLE S1. Taxa used in SSU rDNA tree, with accession numbers and lengths.

name in alignment	taxon	accessions	length (bp)
Environmental_DQ310255	Environmental sequence	DQ310255	2019
Environmental_HM749952	Environmental sequence	HM749952	1785
MK131744_Euglenozoa_sp_host_CR1-1	Symbiontida sp. CR1-1	MK131744	2043
MK131725_Euglenozoa_sp_host_SAG_PB-2	Symbiontida sp. SAG_PB-2	MK131725	2050
MK131743_Euglenozoa_sp_host_AK3_5	Symbiontida sp. AK3_5	MK131743	2053
MK131737_Euglenozoa_sp_host_AK2-3	Symbiontida sp. AK2-3	MK131737	2053
MK131736_Euglenozoa_sp_host_AK2-2	Symbiontida sp. AK2-2	MK131736	2047
MK131735_Euglenozoa_sp_host_AK2-1	Symbiontida sp. AK2-1	MK131735	2044
MK131731_Euglenozoa_sp_host_AK1-2	Symbiontida sp. AK1-2	MK131731	2053
MK131730_Euglenozoa_sp_host_AK1-1	Symbiontida sp. AK1-1	MK131730	2053
MK131727_Euglenozoa_sp_host_SAG_PL-1	Symbiontida sp. SAG_PL-1	MK131727	2039
MK131724_Euglenozoa_sp_host_SAG_PB-1	Symbiontida sp. SAG_PB-1	MK131724	2047
MK131729_Euglenozoa_sp_host_SAG_CdC-1	Symbiontida sp. SAG_CdC-1	MK131729	2050
BOP3.3_1_Symbiontida_sp_MT007911	Symbiontida sp. BoP3.3_1	MT007911	1935
BOP3.3_6_Symbiontida_sp_MT007912	Symbiontida sp. BoP3.3_6	MT007912	1924
KSa7_Symbiontid_cDNA_MT007875	Symbiontida sp. KSa7	MT007875	2048
HLA3_Symbiontida_sp_MT007913	Symbiontida sp. HLA3	MT007913	1874
CBA2_Bihospites_bacati_MT007880	Bihospites bacati	MT007880	1980
Bihospites_bacati_HM004353	Bihospites bacati	HM004353	2056
Bihospites_bacati_HM004354	Bihospites bacati	HM004354	2057
Calkinsia_aureus_EU753419	Calkinsia aureus	EU753419	2034
AB781588_Azumiobodo_hoyamushi	Azumiobodo hoyamushi	AB781588	2074
AB948221_Hemistasia_phaeocysticola	Hemistasia phaeocysticola	AB948221	2020
AF208880_Cryptobia_helicis	Cryptobia helicis	AF208880	2029
AF208888_Bodo_sorokini	Bodo sorokini	AF208888	1995
AF464896_Bodo_designis_strain_DH	Bodo designis	AF464896	2100
AY268046_Klosteria_bodomorphis	Klosteria bodomorphis	AY268046	2072
AY425021_Cryptaulaxoides-like_sp_TCS-2003	Cryptaulaxoides-like sp.	AY425021	2158
AY490221_Bodo_celer	Bodo celer	AY490221	2085
AY490231_Bodo_saltans	Bodo saltans	AY490231	2135
AY665087_Uncultured_eukaryote_clone_SCM15C6	Environmental sequence SCM15C6	AY665087	2016
AY998652_Neobodo_designis_strain_SCCAP_BD56	Neobodo designis	AY998652	2098
DQ207591_Parabodo_caudatus_strain_HFCC10	Parabodo caudatus	DQ207591	2054
DQ207594_Rhynchobodo_sp_HFCC304	Rhynchobodo sp	DQ207594	2137
DQ207598_Rhynchomonas_nasuta_strain_HFCC319	Rhynchomonas nasuta	DQ207598	2152
DQ394362_Sergeia_podlipaevi_strain_CER3	Sergeia podlipaevi	DQ394362	2115
KC205995_Herpetomonas_puellarum_isolate_MCZ-08	Herpetomonas puellarum	KC205995	2141
KC208028_Ichthyobodo_necator_isolate_DK	Ichthyobodo necator	KC208028	2019
KF054131_Blechomonas_keelingi_isolate_B100	Blechomonas keelingi	KF054131	2172
KF633466_Diplonema_papillatum_strain_ATCC_50162	Paradiplonema papillatum	KF633466	2054
KF963538_Paratrypanosoma_confusum_isolate_CUL13	Paratrypanosoma confusum	KF963538	2179

KJ760279_Uncultured_eukaryote_clone_SGYS694	Environmental sequence SGYS694	KJ760279	2063	
KJ762486_Uncultured_eukaryote_clone_SGYH1072	Environmental sequence SGYH1072	KJ762486	2062	
KY947155_Diplonemida_sp_47_RG-2016	Eupelagonemida sp. 47 RG-2016 (SAG)	KY947155	2069	
L14840_Trypanoplasma_borreli_Pg-JH	Trypanoplasma borreli	L14840	2025	
M31432_Trypanosoma_cruzi	Trypanosoma cruzi	M31432	2319	
X07773_Leishmania_donovani	Leishmania donovani	X07773	2205	
MF422196_Rhynchopus_sp_isolate_YPF1521	Rhynchopus sp. YPF1521	MF422196	1990	
MF422200_Diplonemida_sp_isolate_YPF1611	Diplonemida sp. YPF1611	MF422200	2007	
MF422201_Diplonemida_gen._2_sp_1_AH-2017_isolate_YPF1618	Sulcionema specki	MF422201	2010	
MF422203_Hemistasiidae_sp_isolate_YPF1621	Namystinia karyoxenos	MF422203	2064	
KY947154_Diplonemida_sp_37_RG-2016	Eupelagonema pacifica (37_RG-2016 (SAG))	KY947154	2066	*Eupelagonema pacifica* , Okamoto et al 2018
KJ762758_Uncultured_eukaryote_clone_SGYH841	Environmental sequence SGYH841	KJ762758	2063	
KJ762465_Uncultured_eukaryote_clone_SGYH1032	Environmental sequence SGYH1032	KJ762465	2062	
KJ757576_Uncultured_eukaryote_clone_SGYY747	Environmental sequence SGYY747	KJ757576	2061	
KJ762671_Uncultured_eukaryote_clone_SGYH521	Environmental sequence SGYH521	KJ762671	2064	
KY947157_Diplonemida_sp_4sb_RG-2016	Eupelagonemida sp. 4sb RG-2016 (SAG)	KY947157	2069	
KJ762742_Uncultured_eukaryote_clone_SGYH794	Environmental sequence SGYH794	KJ762742	2068	
KY947150_Diplonemida_sp_3_RG-2016	Eupelagonemida sp. 3 RG-2016 (SAG)	KY947150	2069	
KJ762543_Uncultured_eukaryote_clone_SGYH1232	Environmental sequence SGYH1232	KJ762543	2061	
KJ757646_Uncultured_eukaryote_clone_SGYY858	Environmental sequence SGYY858	KJ757646	2062	
KY947152_Diplonemida_sp_21_RG-2016	Eupelagonemida sp. 21 RG-2016 (SAG)	KY947152	2068	
KJ757736_Uncultured_eukaryote_clone_SGYY994	Environmental sequence SGYY994	KJ757736	2061	
KY947156_Diplonemida_sp_1sb_RG-2016	Eupelagonemida sp. 1sb RG-2016 (SAG)	KY947156	2063	
KJ757388_Uncultured_eukaryote_clone_SGYY441	Environmental sequence SGYY441	KJ757388	2061	
KJ757508_Uncultured_eukaryote_clone_SGYY635	Environmental sequence SGYY635	KJ757508	2066	
KJ757308_Uncultured_eukaryote_clone_SGYY1477	Environmental sequence SGYY1477	KJ757308	2055	
KJ760048_Uncultured_eukaryote_clone_SGYS1504	Environmental sequence SGYS1504	KJ760048	2060	
KJ762626_Uncultured_eukaryote_clone_SGYH409	Environmental sequence SGYH409	KJ762626	2059	
KJ762523_Uncultured_eukaryote_clone_SGYH1151	Environmental sequence SGYH1151	KJ762523	2065	
KJ757377_Uncultured_eukaryote_clone_SGYY424	Environmental sequence SGYY424	KJ757377	2062	
KJ761512_Uncultured_eukaryote_clone_SGYX712	Environmental sequence SGYX712	KJ761512	2066	
KJ757605_Uncultured_eukaryote_clone_SGYY792	Environmental sequence SGYY792	KJ757605	2060	
KX189158_Uncultured_marine_diplonemid_clone_Tara_1638_2	Environmental sequence Tara_1638_2	KX189158	1940	
KJ757678_Uncultured_eukaryote_clone_SGYY906	Environmental sequence SGYY906	KJ757678	2063	
KJ762734_Uncultured_eukaryote_clone_SGYH777	Environmental sequence SGYH777	KJ762734	2065	
KJ757730_Uncultured_eukaryote_clone_SGYY981	Environmental sequence SGYY981	KJ757730	2063	
KJ761007_Uncultured_eukaryote_clone_SGYX1230	Environmental sequence SGYX1230	KJ761007	2061	
KY947153_Diplonemida_sp_27_RG-2016	Eupelagonemida sp. 27 RG-2016 (SAG)	KY947153	2074	
KJ757290_Uncultured_eukaryote_clone_SGYY1441	Environmental sequence SGYY1441	KJ757290	2057	
KJ762718_Uncultured_eukaryote_clone_SGYH719	Environmental sequence SGYH719	KJ762718	2061	
KY947151_Diplonemida_sp_13_RG-2016	Eupelagonemida sp. 13 RG-2016 (SAG)	KY947151	2064	
KJ762687_Uncultured_eukaryote_clone_SGYH593	Environmental sequence SGYH593	KJ762687	2058	
KY947159_Diplonemida_sp_21sb_RG-2016	Eupelagonemida sp. 21sb RG-2016 (SAG)	KY947159	2069	
KJ762788_Uncultured_eukaryote_clone_SGYH912	Environmental sequence SGYH912	KJ762788	2065	

KJ762566_Uncultured_eukaryote_clone_SGYH1325	Environmental sequence SGYH1325	KJ762566	2059
KJ757527_Uncultured_eukaryote_clone_SGYY673	Environmental sequence SGYY673	KJ757527	2063
KJ762516_Uncultured_eukaryote_clone_SGYH1139	Environmental sequence SGYH1139	KJ762516	2060
KJ757619_Uncultured_eukaryote_clone_SGYY817	Environmental sequence SGYY817	KJ757619	2061
KX189123_Uncultured_marine_diplonemid_clone_Tara_908_2	Environmental sequence Tara_908_2	KX189123	1940
KJ762804_Uncultured_eukaryote_clone_SGYH958	Environmental sequence SGYH958	KJ762804	2062
AF290080_Uncultured_marine_euglenozoan_DH148-EKB1	Environmental sequence DH148-EKB1	AF290080	2001
KX189159_Uncultured_marine_diplonemid_clone_Tara_1638_3	Environmental sequence Tara_1638_3	KX189159	1940
KJ762460_Uncultured_eukaryote_clone_SGYH1009	Environmental sequence SGYH1009	KJ762460	2066
KJ757153_Uncultured_eukaryote_clone_SGYY1216	Environmental sequence SGYY1216	KJ757153	2054
KX189132_Uncultured_marine_diplonemid_clone_Tara_960_3	Environmental sequence Tara_960_3	KX189132	1939
KX189163_Uncultured_marine_diplonemid_clone_Tara_1638_9	Environmental sequence Tara_1638_9	KX189163	1939
KX189125_Uncultured_marine_diplonemid_clone_Tara_908_5	Environmental sequence Tara_908_5	KX189125	1940
KX189156_Uncultured_marine_diplonemid_clone_Tara_1599_2	Environmental sequence Tara_1599_2	KX189156	1939
KX189126_Uncultured_marine_diplonemid_clone_Tara_908_10	Environmental sequence Tara_908_10	KX189126	1941
DQ504322_Uncultured_diplonemid_clone_LC22_5EP_18	Environmental sequence LC22_5EP_18	DQ504322	1947
KX189170_Uncultured_marine_diplonemid_clone_Tara_1884_4	Environmental sequence Tara_1884_4	KX189170	1941
KX189169_Uncultured_marine_diplonemid_clone_Tara_1884_2	Environmental sequence Tara_1884_2	KX189169	1940
DQ504349_Uncultured_diplonemid_clone_LC22_5EP_32	Environmental sequence LC22_5EP_32	DQ504349	1932
KX189165_Uncultured_marine_diplonemid_clone_Tara_1645_5	Environmental sequence Tara_1645_5	KX189165	1940
KX189155_Uncultured_marine_diplonemid_clone_Tara_1599_1	Environmental sequence Tara_1599_1	KX189155	1940
KX189137_Uncultured_marine_diplonemid_clone_Tara_960_12	Environmental sequence Tara_960_12	KX189137	1937
KJ757684_Uncultured_eukaryote_clone_SGYY913	Environmental sequence SGYY913	KJ757684	2043
KX189134_Uncultured_marine_diplonemid_clone_Tara_960_5	Environmental sequence Tara_960_5	KX189134	1936
DQ504323_Uncultured_diplonemid_clone_LC22_5EP_19	Environmental sequence LC22_5EP_19	DQ504323	1951
KJ762505_Uncultured_eukaryote_clone_SGYH1117	Environmental sequence SGYH1117	KJ762505	2053
KX189127_Uncultured_marine_diplonemid_clone_Tara_908_13	Environmental sequence Tara_908_13	KX189127	1940
KX189167_Uncultured_marine_diplonemid_clone_Tara_1645_8	Environmental sequence Tara_1645_8	KX189167	1941
KX189144_Uncultured_marine_diplonemid_clone_Tara_960_22	Environmental sequence Tara_960_22	KX189144	1940
KX189128_Uncultured_marine_diplonemid_clone_Tara_908_15	Environmental sequence Tara_908_15	KX189128	1941
KX189124_Uncultured_marine_diplonemid_clone_Tara_908_4	Environmental sequence Tara_908_4	KX189124	1941
KX189146_Uncultured_marine_diplonemid_clone_Tara_960_27	Environmental sequence Tara_960_27	KX189146	1940
KX189157_Uncultured_marine_diplonemid_clone_Tara_1599_3	Environmental sequence Tara_1599_3	KX189157	1940
KX189166_Uncultured_marine_diplonemid_clone_Tara_1645_6	Environmental sequence Tara_1645_6	KX189166	1930
KX189139_Uncultured_marine_diplonemid_clone_Tara_960_14	Environmental sequence Tara_960_14	KX189139	1940
KX189171_Uncultured_marine_diplonemid_clone_Tara_1884_5	Environmental sequence Tara_1884_5	KX189171	1942
KX189151_Uncultured_marine_diplonemid_clone_Tara_960_33	Environmental sequence Tara_960_33	KX189151	1939
KX189149_Uncultured_marine_diplonemid_clone_Tara_960_30	Environmental sequence Tara_960_30	KX189149	1940
KX189129_Uncultured_marine_diplonemid_clone_Tara_908_38	Environmental sequence Tara_908_38	KX189129	1940
KX189142_Uncultured_marine_diplonemid_clone_Tara_960_18	Environmental sequence Tara_960_18	KX189142	1940
KX189150_Uncultured_marine_diplonemid_clone_Tara_960_32	Environmental sequence Tara_960_32	KX189150	1938
KX189138_Uncultured_marine_diplonemid_clone_Tara_960_13	Environmental sequence Tara_960_13	KX189138	1935
DQ504321_Uncultured_diplonemid_clone_LC22_5EP_17	Environmental sequence LC22_5EP_17	DQ504321	1944
AY665088_Uncultured_eukaryote_clone_SCM38C39	Environmental sequence SCM38C39	AY665088	2008

KX189173_Uncultured_marine_diplonemid_clone_Tara_2213_5	Environmental sequence Tara_2213_5	KX189173	1941	
KX189172_Uncultured_marine_diplonemid_clone_Tara_2213_1	Environmental sequence Tara_2213_1	KX189172	1940	
KX189162_Uncultured_marine_diplonemid_clone_Tara_1638_8	Environmental sequence Tara_1638_8	KX189162	1948	
KX189133_Uncultured_marine_diplonemid_clone_Tara_960_4	Environmental sequence Tara_960_4	KX189133	1940	
KJ759743_Uncultured_eukaryote_clone_SGYS1011	Environmental sequence SGYS1011	KJ759743	2064	
KX189136_Uncultured_marine_diplonemid_clone_Tara_960_9	Environmental sequence Tara_960_9	KX189136	1942	
KX189143_Uncultured_marine_diplonemid_clone_Tara_960_19	Environmental sequence Tara_960_19	KX189143	1939	
KX189154_Uncultured_marine_diplonemid_clone_Tara_960_42	Environmental sequence Tara_960_42	KX189154	1943	
KX189148_Uncultured_marine_diplonemid_clone_Tara_960_29	Environmental sequence Tara_960_29	KX189148	1940	
KX189145_Uncultured_marine_diplonemid_clone_Tara_960_24	Environmental sequence Tara_960_24	KX189145	1941	
KX189140_Uncultured_marine_diplonemid_clone_Tara_960_15	Environmental sequence Tara_960_15	KX189140	1942	
KX189131_Uncultured_marine_diplonemid_clone_Tara_960_2	Environmental sequence Tara_960_2	KX189131	1943	
KX189130_Uncultured_marine_diplonemid_clone_Tara_960_1	Environmental sequence Tara_960_1	KX189130	1939	
KX189153_Uncultured_marine_diplonemid_clone_Tara_960_40	Environmental sequence Tara_960_40	KX189153	1939	
KX189152_Uncultured_marine_diplonemid_clone_Tara_960_34	Environmental sequence Tara_960_34	KX189152	1939	
KX189147_Uncultured_marine_diplonemid_clone_Tara_960_28	Environmental sequence Tara_960_28	KX189147	1945	
KX189141_Uncultured_marine_diplonemid_clone_Tara_960_17	Environmental sequence Tara_960_17	KX189141	1945	
KX189160_Uncultured_marine_diplonemid_clone_Tara_1638_4	Environmental sequence Tara_1638_4	KX189160	1939	
Eupelago_E8_NO	Eupelagonemid E8	OR831206	2019	this study
Eupelago_E7_NO	Eupelagonemid E7	OR831207	1994	this study
MF422190_Diplonema_sp_isolate_YPF1604	Diplonema japonicum YPF1604	MF422190	2023	
MF422191_Diplonema_sp_isolate_YPF1605	Diplonema aggregatum YPF1605	MF422191	2014	
AY425009_Diplonema_ambulator_strain_ATCC_50223	Diplonema ambulator strain ATCC 50223	AY425009	2062	
AY425011_Diplonema_sp_ATCC_50225	Diplonema sp. 3 ATCC 50225	AY425011	2067	
MF422193_Diplonema_sp_isolate_YPF1508	Diplonema sp. YPF1508	MF422193	2017	
AF119811_Diplonema_papillatum	Paradiplonema papillatum	AF119811	1981	
AY180037_Uncultured_euglenid	Environmental sequence	AY180037	1743	
AY425012_Diplonema_sp_ATCC_50232	Metadiplonema sp. ATCC 50232	AY425012	2148	
MF422192_Diplonema_sp_isolate_YPF1606	Diplonema aggregatum YPF1606	MF422192	2021	
EU635679_Uncultured_marine_diplonemid_clone_PHC3_D1_01	Environmental sequence PHC3 D1 01	EU635679	1177	
EU635680_Uncultured_marine_diplonemid_clone_PHC3_D1_30	Environmental sequence PHC3 D1 30	EU635680	1176	
FN598401_Uncultured_marine_diplonemid_clone_BS23_H10	Environmental sequence BS23 H10	FN598401	1330	
MF422204_Rhynchopus_humris_isolate_YPF1608	Rhynchopus humris YPF1608	MF422204	1884	
MF422195_Rhynchopus_serpens_isolate_YPF1515	Rhynchopus serpens YPF1515	MF422195	1987	
AY425013_Rhynchopus_sp_ATCC_50226	Rhynchopus euleeides ATCC 50226	AY425013	2032	
AY425014_Rhynchopus_sp_ATCC_50229	Rhynchopus sp. ATCC 50229	AY425014	2014	
AY490209_Rhynchopus_sp_SH-2004-I	Rhynchopus sp. SH-2004-I	AY490209	1995	
AY490210_Rhynchopus_sp_SH-2004-II	Rhynchopus sp. SH-2004-II	AY490210	2008	
AY490211_Rhynchopus_sp_SH-2004-IV	Rhynchopus sp. SH-2004-IV	AY490211	1995	
AF119812_Diplonema_sp_2	Flectonema neradi	AF119812	1975	
MF422199_Lacrimia_lanifica_isolate_YPF1601	Lacrimia lanifica YPF1601	MF422199	2004	
MF422202_Hemistasiidae_sp_isolate_YPF1610	Hemistasiidae sp. YPF1610	MF422202	1995	
KY947158_Diplonemida_sp_9sb_RG-2016	Eupelagonemida sp. 9sb RG-2016 (SAG)	KY947158	2062	
AY425010_Diplonema_sp_ATCC_50224	Flectonema neradi	AY425010	2046	

DQ504319_Uncultured_diplonemid_clone_LC22_5EP_10	Environmental sequence LC22_5EP_10	DQ504319	1390
DQ504350_Uncultured_diplonemid_clone_LC23_5EP_5	Environmental sequence LC23_5EP_5	DQ504350	1917
EU635589_Uncultured_marine_diplonemid_clone_Bi2_D1_22	Environmental sequence Bi2_D1_22	EU635589	1197
EU635590_Uncultured_marine_diplonemid_clone_Ti12_D1_18	Environmental sequence Ti12_D1_18	EU635590	1197
EU635591_Uncultured_marine_diplonemid_clone_R26_D1_95	Environmental sequence R26_D1_95	EU635591	1198
EU635593_Uncultured_marine_diplonemid_clone_Ma126_D1_21	Environmental sequence Ma126_D1_21	EU635593	1200
EU635595_Uncultured_marine_diplonemid_clone_KM4_D1_29	Environmental sequence KM4_D1_29	EU635595	1199
EU635599_Uncultured_marine_diplonemid_clone_BS16_D1_08	Environmental sequence BS16_D1_08	EU635599	1199
EU635603_Uncultured_marine_diplonemid_clone_KM4_D1_13	Environmental sequence KM4_D1_13	EU635603	1199
EU635604_Uncultured_marine_diplonemid_clone_BS16_D1_03	Environmental sequence BS16_D1_03	EU635604	1198
EU635605_Uncultured_marine_diplonemid_clone_DH117_D1_41	Environmental sequence DH117_D1_41	EU635605	1201
EU635607_Uncultured_marine_diplonemid_clone_KM4_D1_21	Environmental sequence KM4_D1_21	EU635607	1200
EU635612_Uncultured_marine_diplonemid_clone_KM4_D1_32	Environmental sequence KM4_D1_32	EU635612	1199
EU635613_Uncultured_marine_diplonemid_clone_Ma115_D1_40	Environmental sequence Ma115_D1_40	EU635613	1200
EU635615_Uncultured_marine_diplonemid_clone_Ma131_D1_04	Environmental sequence Ma131_D1_04	EU635615	1199
EU635617_Uncultured_marine_diplonemid_clone_DH136_D1_32	Environmental sequence DH136_D1_32	EU635617	1199
EU635621_Uncultured_marine_diplonemid_clone_R26_D1_77	Environmental sequence R26_D1_77	EU635621	1197
EU635622_Uncultured_marine_diplonemid_clone_DH136_D1_13	Environmental sequence DH136_D1_13	EU635622	1198
EU635624_Uncultured_marine_diplonemid_clone_DH136_D1_55	Environmental sequence DH136_D1_55	EU635624	1200
EU635625_Uncultured_marine_diplonemid_clone_BS16_D1_41	Environmental sequence BS16_D1_41	EU635625	1198
EU635626_Uncultured_marine_diplonemid_clone_KM4_D1_30	Environmental sequence KM4_D1_30	EU635626	1199
EU635627_Uncultured_marine_diplonemid_clone_Ma126_D1_20	Environmental sequence Ma126_D1_20	EU635627	1198
EU635629_Uncultured_marine_diplonemid_clone_DH136_D1_18	Environmental sequence DH136_D1_18	EU635629	1199
EU635630_Uncultured_marine_diplonemid_clone_R26_D1_33	Environmental sequence R26_D1_33	EU635630	1201
EU635631_Uncultured_marine_diplonemid_clone_KM4_D1_42	Environmental sequence KM4_D1_42	EU635631	1198
EU635637_Uncultured_marine_diplonemid_clone_KM4_D1_31	Environmental sequence KM4_D1_31	EU635637	1198
EU635639_Uncultured_marine_diplonemid_clone_R26_D1_40	Environmental sequence R26_D1_40	EU635639	1198
EU635640_Uncultured_marine_diplonemid_clone_Ti11_D1_21	Environmental sequence Ti11_D1_21	EU635640	1201
EU635642_Uncultured_marine_diplonemid_clone_DH117_D1_43	Environmental sequence DH117_D1_43	EU635642	1199
EU635643_Uncultured_marine_diplonemid_clone_DH117_D1_22	Environmental sequence DH117_D1_22	EU635643	1199
EU635645_Uncultured_marine_diplonemid_clone_DH117_D1_24	Environmental sequence DH117_D1_24	EU635645	1199
EU635648_Uncultured_marine_diplonemid_clone_Ma126_D1_26	Environmental sequence Ma126_D1_26	EU635648	1199
EU635655_Uncultured_marine_diplonemid_clone_R26_D1_86	Environmental sequence R26_D1_86	EU635655	1199
EU635657_Uncultured_marine_diplonemid_clone_DH136_D1_66	Environmental sequence DH136_D1_66	EU635657	1199
EU635661_Uncultured_marine_diplonemid_clone_Ti7_D1_24	Environmental sequence Ti7_D1_24	EU635661	1198
EU635665_Uncultured_marine_diplonemid_clone_Ti7_D1_20	Environmental sequence Ti7_D1_20	EU635665	1198
EU635666_Uncultured_marine_diplonemid_clone_Ma136_D1_05	Environmental sequence Ma136_D1_05	EU635666	1200
EU635668_Uncultured_marine_diplonemid_clone_Ma115_D1_42	Environmental sequence Ma115_D1_42	EU635668	1199
EU635669_Uncultured_marine_diplonemid_clone_Ma126_D1_46	Environmental sequence Ma126_D1_46	EU635669	1199
EU635671_Uncultured_marine_diplonemid_clone_Ma131_D1_37	Environmental sequence Ma131_D1_37	EU635671	1199
EU635672_Uncultured_marine_diplonemid_clone_Ma126_D1_39	Environmental sequence Ma126_D1_39	EU635672	1200
EU635678_Uncultured_marine_diplonemid_clone_Ma126_D1_32_DSPDII	Environmental sequence Ma126_D1_32	EU635678	1199
FJ000255_Uncultured_eukaryote_clone_SW1D09	Environmental sequence SW1D09	FJ000255	1566
FJ000260_Uncultured_eukaryote_clone_SW1F04	Environmental sequence SW1F04	FJ000260	1351

FJ000261_Uncultured_eukaryote_clone_452D08	Environmental sequence 452D08	FJ000261	1611
FJ032684_Uncultured_marine_diplonemid_clone_Ma131_1A46	Environmental sequence Ma131_1A46	FJ032684	1917
FN598247_Uncultured_marine_diplonemid_clone_BIO2_F12	Environmental sequence BIO2_F12	FN598247	1349
FN598248_Uncultured_marine_diplonemid_clone_BIO2_F3	Environmental sequence BIO2_F3	FN598248	1348
FN598250_Uncultured_marine_diplonemid_clone_BIO2_H4	Environmental sequence BIO2_H4	FN598250	1348
FN598252_Uncultured_marine_diplonemid_clone_BIO3_A2	Environmental sequence BIO3_A2	FN598252	1343
FN598282_Uncultured_marine_diplonemid_clone_BIO7_B10	Environmental sequence BIO7_B10	FN598282	1341
FN598297_Uncultured_marine_diplonemid_clone_BIO9_B5	Environmental sequence BIO9_B5	FN598297	1344
FN598435_Uncultured_marine_diplonemid_clone_BS3_D7	Environmental sequence BS3_D7	FN598435	1350
FN598436_Uncultured_marine_diplonemid_clone_BS3_D8	Environmental sequence BS3_D8	FN598436	1349
GU820768_Uncultured_euglenozoan_clone_BCA3F14RJ1A09	Environmental sequence BCA3F14RJ1A09	GU820768	1616
JN542573_Uncultured_diplonemid_clone_Kryos_IF_D3	Environmental sequence Kryos_IF_D3	JN542573	1268
KJ760020_Uncultured_eukaryote_clone_SGYS1471	Environmental sequence SGYS1471	KJ760020	2059
KJ762701_Uncultured_eukaryote_clone_SGYH633	Environmental sequence SGYH633	KJ762701	2064
KX189135_Uncultured_marine_diplonemid_clone_Tara_960_6	Environmental sequence Tara_960_6	KX189135	1941
KX189161_Uncultured_marine_diplonemid_clone_Tara_1638_5	Environmental sequence Tara_1638_5	KX189161	1940
KX189164_Uncultured_marine_diplonemid_clone_Tara_1645_4	Environmental sequence Tara_1645_4	KX189164	1920
KX189168_Uncultured_marine_diplonemid_clone_Tara_1645_11	Environmental sequence Tara_1645_11	KX189168	1638
EU635673_Uncultured_marine_diplonemid_clone_Ma115_D1_13_DSPDII	Environmental sequence Ma115_D1_13	EU635673	1197
EU635674_Uncultured_marine_diplonemid_clone_Ma121_D1_12_DSPDII	Environmental sequence Ma121_D1_12	EU635674	1662
EU635675_Uncultured_marine_diplonemid_clone_Bi2_D1_28_DSPDII	Environmental sequence Bi2_D1_28	EU635675	1196
EU635676_Uncultured_marine_diplonemid_clone_Bi2_D1_30_DSPDII	Environmental sequence Bi2_D1_30	EU635676	1206

TABLE S2. Taxa used in multigene analyses, with numbers of genes, percentage of sites covered, and source.

name in alignment	taxon	# genes	% available sites	source 1	source 2
Andagodo	Andalucia godoyi	121	93.5	Leger/Roger In House	PhyloFisher DB
ApicSpir	Apiculatamorpha spiralis PhF-6	125	97.0	PRJNA549754	Tikhonenkov et al 2021
Artemidia	Artemidia motanka	125	99.0	SRR8676455	Kaur et al 2020
Bodosalt	Bodo saltans	115	90.5	GCA_001460835.1	PhyloFisher DB
Diplambu	Diplonema ambulator	124	97.4	SRX3153884	PhyloFisher DB
DiplonemaJ	Diplonema japonicum	125	99.7	SRR8676453	Kaur et al 2020
DiplonemaP	Paradiplonema papillatum	125	99.4	SRR14933372	
EU19	SAG EU19 Zahonova	53	38.4	PRJNA379597	Záhonová et al 2021
Euglgrac	Euglena gracilis	125	98.7	SRR3195326	PhyloFisher DB
Eugllong	Euglena longa	125	99.0	GGOE00000000.1	PhyloFisher DB
EupelE7	Eupelagonemid E7	54	28.1	this study	SRR26871264
EupelE8	Eupelagonemid E8	36	17.9	this study	SRR26871263
Eutrgymn	Eutreptiella gymnastica NIES 381	115	87.5	SRR1294408	PhyloFisher DB
Flecnera	Flectonema neradi	122	95.4	SRX3153887	PhyloFisher DB
Hemistasia	Hemistasia phaeocysticola	125	98.0	SRR9330211	Butenko et al 2020
HeterBB2	Heterolobosean sp. BB2	122	88.5	SRR4291404	PhyloFisher DB
Jakolibe	Jakoba libera	49	25.8	PRJNA12805	PhyloFisher DB
Lacrimia	Lacrimia lanifica	125	98.9	SRR8676452	Kaur et al 2020
Leismajo	Leishmania major	124	98.3	TriTrypDB release 41	PhyloFisher DB
Leptpyrr	Leptomonas pyrrhocoris	125	98.8	TriTrypDB release 41	PhyloFisher DB
Naeggrub	Naegleria gruberi	120	92.1	GCF_000004985.1	PhyloFisher DB
Namystynia	Namystynia karyoxenos	124	98.6	SRR8676451	Kaur et al 2020
Neobdesi	Neobodo designis	113	89.4	https://www.imicrobe.	PhyloFisher DB
PapuAnka	Papus ankaliazontas PhM-4	124	97.7	PRJNA549754	Tikhonenkov et al 2021
Paraconf	Paratrypanosoma confusum	112	91.4	TriTrypDB release 41	PhyloFisher DB
Pharkirb	Pharyngomonas kirbyi	124	97.2	PRJNA301448 (combin	PhyloFisher DB
Reclamer	Reclinomonas americana	92	52.4	TBestDB	PhyloFisher DB
RhynchoE	Rhynchopus euleeides	125	99.2	SRR5998382	
RhynchoH	Rhynchopus humris	125	99.5	SRR8676454	Kaur et al 2020

Styginca	Stygiella incarcerata	123	95.5	SRR2566811	PhyloFisher DB
Sulcionema	Sulcionema specki	125	99.4	SRR8676450	Kaur et al 2020
Trypbruc	Trypanomonas brucei	124	98.3	GCF_000210295.1	PhyloFisher DB
Tsukglob	Tsukubamonas globosus	88	46.2	DRR014073	PhyloFisher DB
